# A Mixture Approach to Vagueness and Ambiguity

**DOI:** 10.1371/journal.pone.0063507

**Published:** 2013-05-07

**Authors:** Steven Verheyen, Gert Storms

**Affiliations:** Faculty of Psychology and Educational Sciences, University of Leuven, Leuven, Belgium; University of Leicester, United Kingdom

## Abstract

When asked to indicate which items from a set of candidates belong to a particular natural language category inter-individual differences occur: Individuals disagree which items should be considered category members. The premise of this paper is that these inter-individual differences in semantic categorization reflect both ambiguity and vagueness. Categorization differences are said to be due to ambiguity when individuals employ different criteria for categorization. For instance, individuals may disagree whether *hiking* or *darts* is the better example of sports because they emphasize respectively whether an activity is strenuous and whether rules apply. Categorization differences are said to be due to vagueness when individuals employ different cut-offs for separating members from non-members. For instance, the decision to include *hiking* in the sports category or not, may hinge on how strenuous different individuals require sports to be. This claim is supported by the application of a mixture model to categorization data for eight natural language categories. The mixture model can identify latent groups of categorizers who regard different items likely category members (i.e., ambiguity) with categorizers within each of the groups differing in their propensity to provide membership responses (i.e., vagueness). The identified subgroups are shown to emphasize different sets of category attributes when making their categorization decisions.

## Vagueness and Ambiguity

Many - if not most - of the words we regularly use are vague. Although we are familiar with their meaning, we can at times be uncertain whether they apply to a particular instance or not. Adjectives like tall and bald are textbook examples [1]–[3]. We have no difficulty distinguishing women who are clearly tall from women who are clearly not, but might find it difficult to say whether a woman who is slightly above average height is tall or not. A man with zero hairs is definitely bald and a man with a full head of hair is definitely not. Many a hairdo resides at the boundary of bald and not bald though.

Vagueness is not restricted to adjectives. Nouns too pick out categories with vague boundaries, which leaves the membership status of some instances unclear [4]–[10]. Perhaps the example that comes easiest to mind is that of the *tomato*. Is it a vegetable or is it not? Research in psychology has shown that individuals answer this question differently, despite having all indicated that they know the meaning of the terms involved [11], [12]. Inter-individual differences in the answer to category membership questions like these have become an important source of information about the vagueness of words, dating back to the nineteen-thirties [13].

Both in philosophy and psychology the vagueness of language terms is generally thought to result from individuals adopting different cut-offs in separating category members from non-members [3]. According to these prevailing views a word like tall is vague because individuals differ with respect to the height from which they start to call women tall. While one individual may require a woman to be 175 cm in height to be termed tall, someone else may require a woman to be at least 180 cm. In this example, one would of course expect that the proportion of individuals who call a woman tall increases with her height. Typically, candidate instances for a vague word can be organized along a particular dimension of variation (e.g., height), with the word being endorsed more often the further an instance is positioned along the relevant dimension.

In treatments of vagueness one often presumes to know the dimension of variation along which the cuts are made. For tall it might appear trivial that one would use height as a criterion. For bald the issue is already less trivial. One could suggest the number of hairs on one's scalp as a criterion for determining whether someone is rightfully called bald or not. However, the position of the hairs along the scalp and the manner in which the hairs are organized might also matter (see the lengths to which some men go to construct elaborate comb-overs). The issue is even more pronounced for nouns. Membership in noun categories is not determined by necessary and sufficient criteria, but is based on a number of attributes that are merely characteristic of the category [14]. Wittgenstein's treatment of games has become the paradigmatic example [15]. Games share attributes, but there is not one attribute they all have in common. Many games have a competitive element, for instance, but not all (e.g., *solitaire*). Like most games, *solitaire* does come with rules and is played for amusement. Instances are regarded better examples of a category, the more of these shared characteristic attributes they possess [16], [17].

The existence of several dimensions of variation opens the door to a notion that is related to, but different from vagueness: ambiguity [3], [18], [19]. Ambiguity arises when individuals employ different criteria to determine whether a word applies to an instance or not. For instance, a difference of opinion as to whether a man is truly bald is the result of ambiguity, not vagueness when one judge uses the number of hairs as a criterion and the other the distribution of hairs across the scalp. Note that the resolution of ambiguity does not resolve vagueness. Even if one agrees that the number of hairs should be used as the criterion for baldness, vagueness may persist when individuals do not agree on the required number of hairs.

The example above employs two dictionary senses of bald (<lacking hair on the head> and <lacking a natural or usual covering>), but ambiguity may be more subtle. The literature on noun categories contains ample evidence that even one's current goals or interests and recent or typical interactions with instances of a category can affect what attributes are accentuated in semantic categorization [20]–[23]. As a result it has proven notoriously difficult to disentangle vagueness and ambiguity [24]. Many accounts of vagueness therefore shy away from the problem by presuming the dimension of variation along which the cuts are made to be known or by leaving it unspecified. That is, they prefer to focus on how vagueness may persist when everyone is assumed to use the same criteria to judge whether a word applies to a particular instance.

In what follows we too want to propose an account of vagueness, but one that does not discard ambiguity from the start. Rather, the account allows for the identification of groups of individuals who employ different dimensions of variation to judge whether a word applies to a particular instance or not. Within each of the identified groups vagueness is thought to arise from individuals adopting different cut-offs along a common dimension of variation. Both in the exposition of the account and in its application we have decided to focus on noun categories because, as was noted above, the vagueness-ambiguity issue seems more pronounced for these parts of speech. The application to other word classes is straightforward, however. As we will see, for the approach to work one only requires categorization decisions towards a set of candidate instances that elicit considerable inter-individual differences. Such borderline items constitute the natural choice of materials in any study on vagueness.

## Introduction to the Approach

Imagine we asked a number of individuals to judge whether the label sports applies to a set of candidate items. To start off, we assume that there is no ambiguity in play, only vagueness. That is, everyone uses the same criteria to decide on category membership. The use of different cut-offs to separate members from non-members is the only source of inter-individual differences in categorization. Say all respondents require candidate instances to be activities that are physically demanding. Certain individuals may want to see more evidence of this requirement than others, though. Seeing that *hiking* is physically more demanding than *darts* is, some respondents might only deem *hiking* physical enough to be considered a sport, while others might find both *darts* and *hiking* demanding enough. Across all respondents one would expect *hiking* to be more often endorsed than *darts* as it meets the category requirements better.

It is clear that in this hypothetical example the individuals' response patterns are informative with respect to the dimension of variation along which the cuts are made. Notably, the responses of any individual would follow a Guttman structure if they were arranged according to the proportion of individuals who endorsed them as category members. A Guttman structure with *n* entries consists of a series of *k* zeros, followed by a series of *n*–*k* ones (e.g., 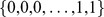). The order of instances is invariant across individuals. It suggests that all individuals employ the same criteria to decide whether to endorse an instance or not, with a higher probability of being endorsed, the better an instance meets the requirements. The value of *k* may differ between individuals, suggesting we are dealing with a vague concept without generally agreed upon cut-off between members and non-members. For instance, patterns 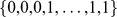 and 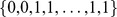 would indicate that the first respondent imposes a higher requirement than the second respondent does. In our hypothetical example we presumed that all individuals were judging category membership based on how demanding an activity is. The more demanding activities would then be placed more to the right if activities were to be arranged according to the proportion of individuals who endorsed them as sports. If one were not to know beforehand the dimension of variation individuals were using to judge category membership, one could thus infer it from the Guttman structure by establishing that activities are positioned more to the right (are endorsed more often) the more demanding they are.

Let us now assume that the concept of sports is not only vague, but also ambiguous. For instance, among the respondents there are those who feel a sport is an activity that is physically demanding and those who place more emphasis on elements such as rules or competition. These distinct views on what are considered representative category members are expected to result in marked categorization differences between the groups, because instances that satisfy some requirements, do not necessarily satisfy others. Among the former individuals *hiking* is expected to be more frequently endorsed as an example of sport than *darts* is, seeing that it is the physically more demanding activity. Among the latter individuals *darts* is expected to be more frequently endorsed than *hiking* is since the rule and competition requirements are better met by *darts* than by *hiking*. Disagreements like the one whether *hiking* or *darts* is the more likely sport can thus be capitalized on to identify ambiguity. In groups that take distinct views on category membership, arranging the candidate instances according to categorization proportions is likely to yield different organizations with different interpretations of the dimensions of variation used to decide on category membership.

Of course, when one has actual categorization decisions at one's disposal one does not know the extent to which the inter-individual differences in categorization are the result of vagueness and ambiguity. One does not know whether there are individuals who employ different criteria for categorization or who employs which criteria. One would like to check the respondent sample for the existence of *latent* groups, with the understanding that individuals within a group display consistent categorization behavior (i.e., share the same criteria) that is different from the categorization behavior of other groups (i.e., they employ different criteria). Mixture models are appropriate tools to accomplish this. Mixture models are statistical models for representing the presence of sub-populations within an overall population, without requiring that the observed data set should identify the subpopulation to which an individual belongs [25].

The mixture modeling framework we propose allows one to partition a participant sample in a number of latent groups that are different in terms of the dimension of variation they use to judge category membership (i.e., ambiguity). To do so it capitalizes on the insight that the use of different criteria is likely to result in different organizations of the candidate instances (see above). Contrary to our hypothetical example, however, the different organizations of candidate instances are arrived at without prior knowledge of the criteria that are in use in the participant sample or any other a priori division of the participants. Instead, they are inferred from the data. Participants who are placed together in a group are consequently understood to use the same criteria for categorization. These categorizers can, however, display varying degrees of propensity to endorse items as category members (i.e., vagueness).

We will use the mixture approach to verify whether earlier approaches have justly discarded ambiguity when accounting for the vagueness of natural language categories. When our approach suggests a categorization data set is best accounted for by a single group, there is no evidence for ambiguity. All the inter-individual categorization differences are then due to vagueness. When a solution with more than one group is retained, this constitutes evidence for ambiguity in addition to vagueness. Categorization differences between individuals in the same group are believed to be due to vagueness. Categorization differences between individuals from different groups are believed to be due to ambiguity.

The goal of Study 1 is to establish the extent to which vagueness and ambiguity are responsible for the inter-individual differences present in an apparently homogeneous sample of categorizers. Foreshadowing our main result, it will allow us to demonstrate that contrary to what is customarily assumed these inter-individual differences are not only due to vagueness, but to ambiguity as well. With the identification of latent groups of categorizers who employ different criteria for categorization, the nature of these criteria differences (i.e., the dimensions of variation employed) are not yet identified, however. As we already mentioned, it is straightforward to investigate these differences by inspecting the relative positions of the instances and relating these positions to external information one might have about the instances. The goal of Study 2 is therefore to uncover the nature of the between-group categorization differences. The different subgroups will be shown to emphasize different sets of category attributes when making their categorization decisions. Before turning to the details of Studies 1 and 2, we will elaborate on the formal details of our approach.

## Model Details

From the general class of mixture models we propose to use a mixture item response theory model [26], [27]. Mixture item response theory models are traditionally employed to assess individuals' aptitudes and dispositions in response to a number of test items. However, the one-group variant has also been applied to semantic categorization [28], [29]. With traditional approaches to vagueness this particular model has in common that it discards ambiguity from the start and presumes that only vagueness is into play. That is, it assumes that all individuals command the same category requirements and differ only in the degree to which it needs to be expressed in an instance to endorse it as a category member. Thus, participants do not differ in terms of the criteria they use, but may do so in the categorization cut-off they employ. This particular model uses the information that is contained in the individuals' response patterns to organize both individuals and instances along a latent dimension, much like the procedure that was outlined for our hypothetical example did. The main difference with this earlier model is that in the mixture model the assumption that all participants adhere to the same dimension is relaxed. Instead, it is assumed that the participants divide in subgroups with a different organization of instances each. Within each subgroup, individuals are still thought to differ in terms of the employed categorization cut-off. The model in Equation (1), then, is a mixture of differently parameterized vagueness-only models of the kind that has already been applied to semantic categorization [28], [29]. It allows for ambiguity in addition to vagueness.

Binary categorization decisions 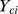 constitute the input for the mixture model. 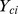 takes value 1 when categorizer *c* decides that instance *i* is a member of the target category and takes value 0 when *c* decides that *i* is not a member. Every one of these categorization decisions is considered the outcome of a Bernoulli trial with the probability of a positive categorization response:
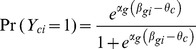
(1)


The model in Equation (1) organizes the candidate instances along a dimension according to their likelihood of being endorsed. A separate dimension is extracted for each group *g* of categorizers that is inferred from the data. 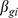 indicates the position of instance *i* along the dimension for group *g*. Higher values for 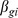 indicate instances that are more likely to be endorsed. It is assumed that individuals in a group employ the same criteria, and that the organization of the instances can thus be conceived of as reflecting the extent to which they meet these requirements. The better an instance meets the criteria, the more likely it is to be endorsed and consequently the higher its 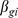 estimate.

Groups with different criteria will value different attributes in instances, which in turn will affect the relative likelihood with which various instances are endorsed. The model therefore identifies subgroups that require separate 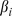 estimates. An instance *i* that meets many of the criteria of group *g* will often be selected by the members of *g*, resulting in a high 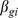 estimate. The same instance might just meet a couple of the criteria of a different group *g'*. As *i* will then not be endorsed by the members of *g'* the estimate of 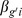 will be low.

Individuals who employ the same criteria may still differ regarding the number of instances that make up their selection, depending on the cut-off they use in separating members from non-members. They may select a large or small number of items, depending on whether they require instances to meet the requirements to a small or to a large extent, respectively. Above, we identified the latent dimension with the membership criteria and the positions of instances along the dimension with the extent to which they meet these criteria. In a similar vein, individuals are awarded a position along the dimension, indicating the extent to which they require instances to meet the criteria in order to be endorsed. In Equation (1) 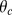 indicates the position of categorizer *c* along the dimension for the group *c* is placed in. With the positions of the instances fixed for all categorizers that belong to the same group, high 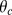 estimates (i.e., high standards) correspond to small extensions, while low 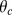 estimates (i.e., low standards) correspond to large extensions. The 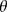
's in Equation (1) thus capture the degree of liberalness/conservatism categorizers display.

The model in Equation (1) is a probabilistic one. It requires that individuals' response patterns have a probabilistic Guttman structure. That is, an instance that is positioned to the right of the cut-off will not necessarily be endorsed as a category member. Neither does a position to the left of the cut-off imply that the instance will definitely not be endorsed as a category member. Each categorization decision is considered the outcome of a Bernoulli trial with the probability of a positive categorization response determined by the relative position of instance and cut-off. An instance is more likely to be endorsed as a category member the more to the right of the cut-off it is positioned. The more to the left of the cut-off an instance is positioned, the less likely it will be endorsed. Across respondents the probability of selection increases from left to right. A separate 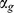 for each group determines the shape of the response function that relates the unbounded extent to which an object surpasses/falls short of the cut-off (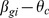) to the probability of categorization (bounded between 0 and 1). Unlike the 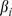
's and the 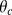
's, the 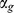
's in Equation (1) can only take on positive values.

## Study 1: Modeling Inter-Individual Differences in Categorization

In Study 1 we will revisit the semantic categorization data the vagueness-only model has already been applied to [29], using the mixture version of the model in Equation (1). We will consider solutions with 1, 2, 3, 4, and 5 latent subgroups. The one-group solution is the equivalent of the vagueness-only analysis that has already been undertaken. For there to be evidence in favor of both ambiguity and vagueness, an analysis of the categorization data with the mixture model would have to yield at least two subgroups of categorizers.

### Method

#### Ethics statement

Study 1 was conducted with the approval of the review board of the University of Leuven. Written informed consent was obtained from all participants.

#### Participants

Two hundred and fifty first year psychology students at the University of Leuven completed a categorization task as part of a course requirement.

#### Materials

The materials consisted of 8 categories with 24 items each. The categories included two animal categories (fish and insects), two artifact categories (furniture and tools), two borderline artifact-natural-kind categories (fruits and vegetables), and two activity categories (sciences and sports). The corresponding items included both clear members, clear non-members, and borderline cases. Note that throughout the text we will continue to employ an italic typeface to denote items and a small capital typeface to denote categories.

#### Procedure

Each of the participants was handed an eight page questionnaire to fill out. They were told to carefully read through the 24 items on each page and to decide for each item whether or not it belonged in the category printed on top of the page. Participants indicated their answer by either circling 1 for *member* or 0 for *non-member*. Five different orders of category administration were combined with 2 different orders of item administration, resulting in 10 different questionnaires. Each of these was filled out by 25 participants. The categorization data are available for download from the first author's website: http://ppw.kuleuven.be/concat/.

#### Model analyses

Each category's categorization data were analyzed separately using the model in Equation (1). For every category solutions with 1, 2, 3, 4, and 5 latent subgroups were obtained. This was done using WinBUGS [30] following the procedures that have been outlined for the Bayesian estimation of mixture item response theory models [31]. These include suggestions for the specification of the priors for the model parameters, which we adopted:
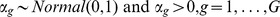


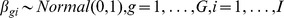


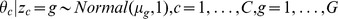


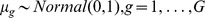


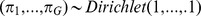


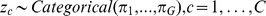
with *G* the number of latent groups (1 to 5), *I* the number of candidate items (24 for each category) and *C* the number of categorizers (250 for each category). Latent group membership was parameterized as a multinomially distributed random variable with 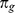 reflecting the probability of membership in subgroup *g*. 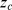 is the latent variable that does the group assignment.

The results are based on 3 chains of 10,000 samples each, with a burn-in of 4,000 samples. The chains were checked for convergence and label switching. All reported values are posterior means, except for group membership which is based on the posterior mode of 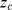.

### Results

#### Model comparisons

When determining the required number of latent groups, both fit and complexity need to be considered [32]. With additional subgroups come additional parameter estimates. That might provide for an improvement in fit, but not necessarily for a better understanding. The resulting account might end up being too complex for the data. To determine the suitable number of latent groups we will rely upon the Bayesian Information Criterion (BIC). The BIC provides an indication of the balance between goodness-of-fit and model complexity for every solution [33]. Results of a recovery study have shown that the BIC can be used to choose among mixture solutions with a different number of subgroups [31]. The solution to be preferred is that with the lowest BIC. Table 1 holds for every category five BIC values, corresponding to five partitions of increasing complexity. For each category the lowest BIC is set in bold typeface.

**Table 1:** BIC values for five partitions of the categorization data.

	BIC				
Category	1 group	2 groups	3 groups	4 groups	5 groups
fish	3940	**3780**	3794	3971	4096
fruits	**3304**	3464	3613	3762	3911
furniture	**3438**	3602	3750	3586	3735
insects	4546	**4406**	4483	4626	4776
sciences	4398	4140	**4032**	4170	4311
sports	3531	**3290**	3413	3562	3711
tools	3826	**3606**	3726	3873	4020
vegetables	**3490**	3610	3757	3906	4054

There were three categories for which the BIC indicated that a one-group solution was to be preferred. This was the case for fruits, vegetables, and furniture. This suggests that the inter-individual categorization differences for these categories only reflect vagueness. The categorizers employed different cut-offs, but used the same criteria to determine category membership. For the remainder of the categories the BIC indicated that multiple groups could be discerned among the categorizers. In the case of insects, sports, fish, and tools the BIC suggested there were two such groups. In the case of sciences the BIC suggested there were three. Hence, for five of the eight categories there is evidence for ambiguity in addition to vagueness. The categorizers could be partitioned in groups that organized the items differently with respect to the target category. This suggests they employed different criteria for category membership (ambiguity). Within each group the categorizers employed different cut-offs (vagueness).

The participants divided in two groups of about equal size for the categories of sports and tools. In the case of sports one group comprised 51% of the participants. The second group comprised the remaining 49%. In the case of tools these percentages equaled 54 and 46. The same participants divided in a larger and smaller group for the categories of fish and insects. In the case of fish the dominant group comprised 79% of the participants. The second group comprised the remaining 21%. For insects these percentages equaled 72 and 28. The three groups for the sciences category comprised 46%, 31%, and 23% of the participants. The magnitudes of these percentages indicate that the model analyses did not just pick out a few oddly behaving categorizers. The smallest of the groups comprised 52 participants (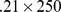 participants in the second fish group). The BIC is a conservative model selection heuristic that heavily penalizes complex models. As such it protects against a large number of groups with a small number of idiosyncratic categorizers each. Since every group comes with 24 additional 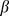 parameters, a considerable number of participants had to employ a different set of criteria for them to end up in a separate group.

#### Group comparisons

With a considerable number of participants in each group, the dimensions of variation employed do not have to differ dramatically. A small reorganization of the items that allows for a better account of the data of a sizeable group of participants, suffices for a more complex model to be chosen. When the goal is to model categorization decisions regarding natural language categories this is a desirable property. After all, we do not expect individuals to have radically different ideas about the extension of these categories. That would seriously hamper daily communication. The correlation between the mean 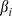 estimates from each group gives an indication of the difference between their dimensions of variation. This correlation equaled 91 for sports and 89 for tools. These numbers clearly illustrate that a small difference in item organization may suffice for a group to become separated when this group is sizeable enough. (The participants divided in two groups of about equal size for the categories of sports and tools.) The correlation equaled 73 for fish and.79 for insects. For these categories the participants were divided in a larger and a smaller group. For sciences the correlation between the mean 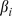 estimates from the largest group and those from the second largest group equaled .77. Both groups correlated .73 with the smallest group.

The correlations between the mean 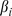 estimates from each group indicate that there is common ground among the language users in the different groups: All the correlations were significant at the 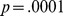 level (one-tailed *t*). This does not come as a surprise. All participants are part of the same language community where they presumably exchange these category terms without experiencing major misunderstandings. Nevertheless, there appeared to be reliable differences regarding their organization of items with respect to familiar categories like sports, tools, fish, insects, and sciences. For each of these categories the participant sample could be partitioned in subgroups of distinctly behaving categorizers. How much the subgroups differed varied from one category to the other, with the smallest difference emerging for the category of sports and the greatest difference emerging for the category of fish. In both categories one can find clear examples of items that were regarded differently by the participants in the two subgroups, however. The larger group for fish considered *whales* 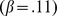 likelier category members than *oysters* 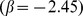, while the smaller group had the opposite opinion 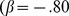 and 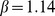, respectively). For sports a similar difference held for the items *hiking* and *darts*, with their respective 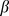
's −1.25 and .38 in one group and .45 and −.95 in the other.

#### Model fit

**Figure 1:**
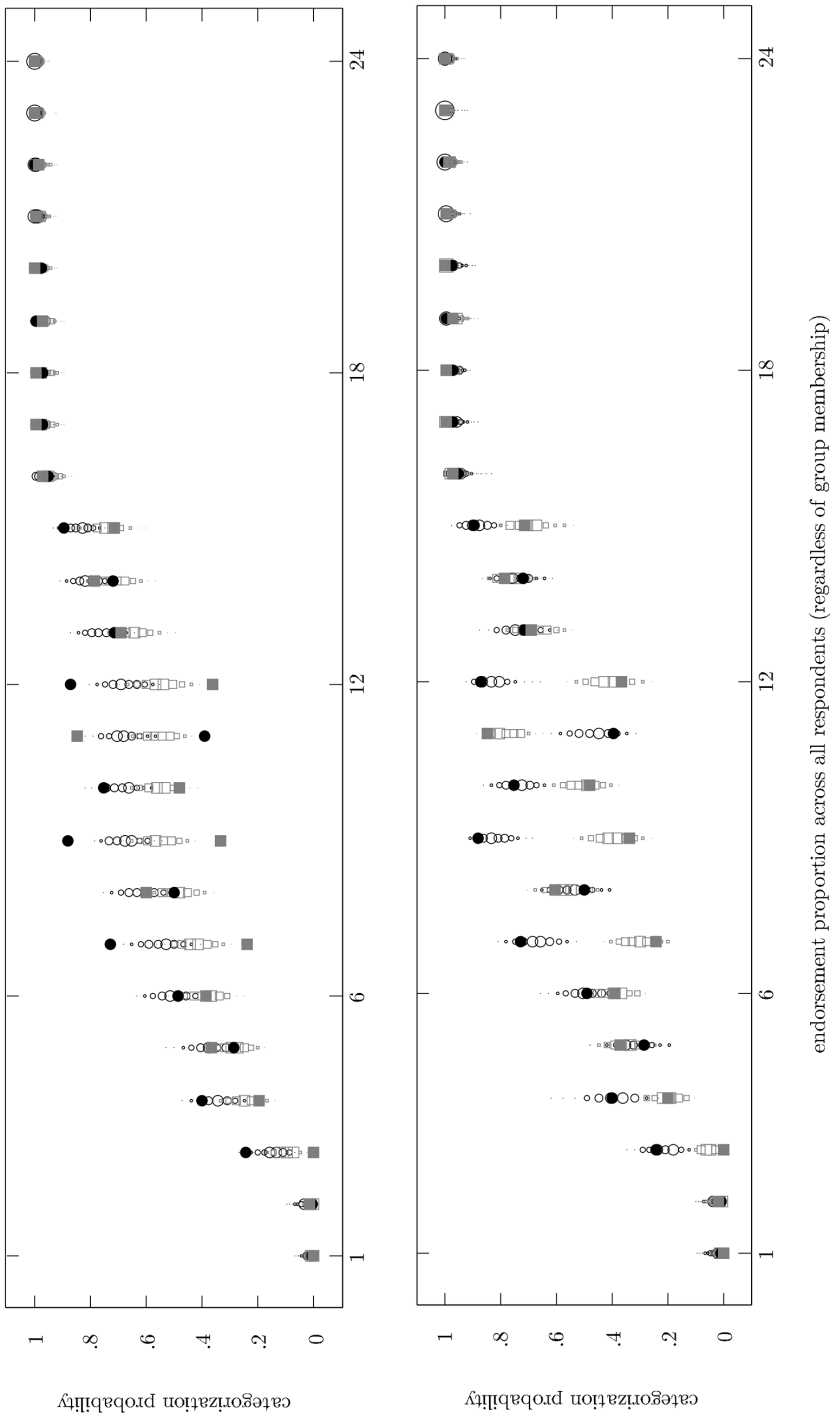
Posterior predictive distribution of the one-group model (upper panel) and the two-group model (lower panel) for the sports categorization data. Items are ordered along the horizontal axes according to the number of participants out of 250 who endorsed them as category members. Filled black circles show per item the proportion of participants from Group 1 who provided a positive categorization response. Filled gray squares show per item the proportion of participants from Group 2 who provided a positive categorization response. Outlines of circles and squares represent the posterior predictive distribution of positive categorization decisions for Group 1 and Group 2, respectively. The size of these outlines is proportional to the posterior mass that is given to the various categorization probabilities.

The BIC is a relative measure of fit. For a given data set it indicates which model from of a set of candidate models is to be preferred in terms of fit and complexity. The BIC is not an absolute measure of fit, however. It doesn't indicate whether the preferred model adequately describes the data it was fitted to. We used the posterior predictive distribution to see whether this was the case. The posterior predictive distribution represents the relative probability of different observable outcomes after the model has been fitted to the data. It allowed us to assess whether the solutions with multiple groups fit the categorization data in absolute terms. In addition, it was insightful to include the posterior predictive distribution for the one-group model to see how it compares with the more complex models. This is illustrated for sports in Figure 1.

In Figure 1 the 24 candidate items are placed along the horizontal axes in increasing order of endorsement (across all 250 respondents). The BIC indicated that for sports the categorizers divided in two groups of equal size. For each item a filled black circle represents the proportion of participants from the first group who provided a positive categorization response. Filled gray squares represent the proportion of participants from the second group who provided a positive categorization response. The two panels in Figure 1 are identical with respect to these data. Whether a positive or a negative response was favored could depend on the group of categorizers. Item 12 (*darts*), for instance, was considered a category exemplar by many of the categorizers in Group 1 (black circle), while many of the categorizers in Group 2 (gray square) did not consider it an exemplar. Similar divergences between the groups occurred for items 7 (*chess*), 9 (*billiards*), and 11 (*hiking*). These notable categorization differences support the division the BIC suggested.

The upper panel in Figure 1 shows the posterior predictive distribution of positive categorization decisions resulting from the one-group model. The lower panel shows the posterior predictive distribution of positive categorization decisions resulting from the two-group model. For every item the panels include a separate distribution for each categorization group (circular outlines for Group 1, square outlines for Group 2). The size of the plot symbols is proportional to the posterior mass given to the various categorization probabilities. It is clear that the one-group model did not capture the group differences in categorization that were identified for items 7, 9, 11, and 12. The model predicted categorization probabilities that were in between the categorization proportions that were observed for the two groups. Because the model adopted the same dimension of variation for both groups, it is not surprising that it could not predict very different outcomes. The two-group model *could* yield different model predictions due to its separate item organization for each group. In the lower panel of Figure 1 the posterior predictive distributions for the two groups are quite different when this was required. In the case of item 12, for instance, a positive categorization response was predicted for the Group 1 members, while negative categorization responses were predicted for the Group 2 members. Figure 1 thus clearly shows that for sports the two-group model provided a better fit to the categorization data than the one-group model did. In addition, inspection of the lower panel of Figure 1 learns that the two-group model's predictions closely mirrored the observed data: The model is appropriate for the data in absolute terms as well. This conclusion holds in all other categories for which the BIC indicated a more complex model was to be preferred.

### Discussion

The results from Study 1 indicate that inter-individual differences in semantic categorization need not only indicate vagueness. They can result from ambiguity as well. While for some natural language categories (fruits, furniture, vegetables) the most parsimonious account of the inter-individual categorization differences involves the use of different cut-offs for common criteria, other categories (fish, insects, sciences, sports, tools) require the additional assumption of the use of different criteria by different participants. In this respect these results qualify those that involved the application of a vagueness-only model to the same categorization data [29]. That particular model is found to apply here, with the understanding that it does so in subgroups of participants who employ different criteria for category membership.

First and foremost this finding has important implications for the so called threshold theory of categorization [34]–[36] of which the vagueness-only model was intended to be a formalization. The threshold theory assumes that prior to categorization respondents assess the similarity between item and category. The position of the item along the latent dimension is thought to reflect the outcome of this assessment, with items that are highly similar to the category receiving a position further along the dimension than items that resemble the category to a lesser degree. This item-category-similarity is then compared against an internal threshold 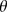, the position of the categorizer along the dimension, to decide whether it affords a positive rather than a negative decision. The existence of multiple item organizations for a single category suggests that it might be improper to assume a default similarity assessment outcome that is the same for all language users. Rather, it would appear that there exist a number of these default outcomes, some of which may be more prominent than others. Which one of these defaults is involved in the categorization responses of a particular participant is then indicated by that participant's group membership, with the size of the group providing an indication of the prominence of the corresponding assessment outcome. For each of the categories the extracted subgroups were few in number and considerable in size, suggesting that the goals, interests, experiences and/or interactions with category instances that might be responsible for the ambiguity, are largely shared by members of the language community, rather than idiosyncratic.

Of course, the finding that for several categories there is more than one dimension of variation that informs categorization has implications for any account of inter-individual categorization differences that presumes this dimension to be known or leaves it unspecified [3]. They run the risk of attributing differences which in fact result from ambiguity to vagueness and/or attributing differences that result from variation along one dimension to variation along another. To obtain a better understanding of the manner in which these dimensions of variation differ, external measures can be related to the 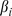 estimates of the different groups. In Study 2 the employed criteria are substantiated using attributes that are deemed characteristic of the target categories.

## Study 2: Substantiating the Criteria for Semantic Categorization

In Study 2 an explanation of the items' positions along the latent dimensions is attempted. The focus will be on the group differences herein since this ambiguity constitutes the cardinal contribution of Study 1. Before, differences like these had only been shown between groups of categorizers that were a priori known to differ in a respect considered important for categorization [21], [22]. The main research question shifts from “Are there group differences in categorization?” in the previous study to “How are the groups different?” in the current study. To answer this question their 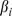 estimates are related to external measures that may reveal the considerations that go into the categorization decisions. First, we determine to what extent attributes that participants consider important for category membership are true of the different candidate items. Then, we obtain a small number of principal components that convey the information that is contained in these attribute applicability judgments. Finally, the item organizations of different groups are regressed upon these principal components to look for distinct patterns of attention to or weighting of the attribute information they represent.

Characteristic category attributes were already available for the categories of fish, fruits, insects, sports, tools, and vegetables [37]. For the purpose of this Study, additional category attributes were collected for furniture and sciences. For each category a matrix was then constructed indicating which category attributes apply to which category items. The applicability matrices for fish, fruits, insects, and vegetables have already been described elsewhere [28]. The applicability matrices for furniture, sciences, sports, and tools are new.

### Method

#### Ethics statement

Study 2 was conducted with the approval of the review board of the University of Leuven. Written informed consent was obtained from all participants.

#### Participants

One hundred and twenty University of Leuven students provided the original category attributes [37]. We recruited 45 additional participants from the same student population. Forty of them provided characteristic category attributes as part of a course requirement. The remaining five students provided attribute applicability judgments. They were paid the equivalent of 10$ per hour.

#### Attribute generation task

For six of the categories from Study 1 characteristic attributes were already available. The attributes were obtained by having 120 University of Leuven students (20 for each category) list those attributes they felt were important for something to be considered a member of the category. Participants were asked to generate up to 10 of these attributes. Only attributes generated toward a category by at least two participants were selected. This resulted in 31, 32, 35, 33, 34, and 29 attributes for the categories of fish, fruits, insects, sports, tools, and vegetables, respectively. This procedure was repeated for furniture and sciences yielding 33 and 39 attributes, respectively. Throughout the text attributes will be printed between triangular brackets in an italic typeface.

#### Attribute applicability judgment task

For each category an applicability matrix was constructed. The rows of a category's applicability matrix are comprised of that category's characteristic attributes. The category's 24 candidate items from Study 1 constitute its columns. Five University of Leuven students indicated for each item-attribute-pair in a matrix whether the attribute applied to the item or not, by entering a 1 or a 0 in the corresponding matrix cell. In case of uncertainty the participants were asked to provide their best guess. Participants performed the task at home and could freely choose when they worked on it. They were given the choice to work on the task row-wise or column-wise, but they were asked not to pause until a row or column was finished. In this manner five applicability matrices were obtained for every category. These matrices were summed and input to a principal components analysis. The matrices are available for download from the first author's website: http://ppw.kuleuven.be/concat/.

### Results

Through a principal components analysis the variation in each summed applicability matrix was described in terms of a limited set of uncorrelated variables (i.e., components). This was achieved by excluding those principal components with eigenvalues less than the average [38]. This criterion yielded 6 components, except for sciences (5) and tools and vegetables (both 7). The variance in the applicability matrices that was accounted for by this selection of components ranged between 76% for sciences and 85% for fish. The resulting components are suitable independent variables in regression analyses that have a category's item organization as a dependent variable: They are not correlated and are relatively small in number compared to the 24 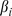 estimates that are regressed upon them. To ascertain which category components contribute to each subgroup's mean 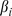 estimates (obtained in Study 1) a Bayesian multiple linear regression [39] was conducted using JAGS [40]. It was decided that for a component to have a credible nonzero contribution the 95% highest density interval (HDI) of the posterior of its regression weight was not to include 0.

**Figure 2:**
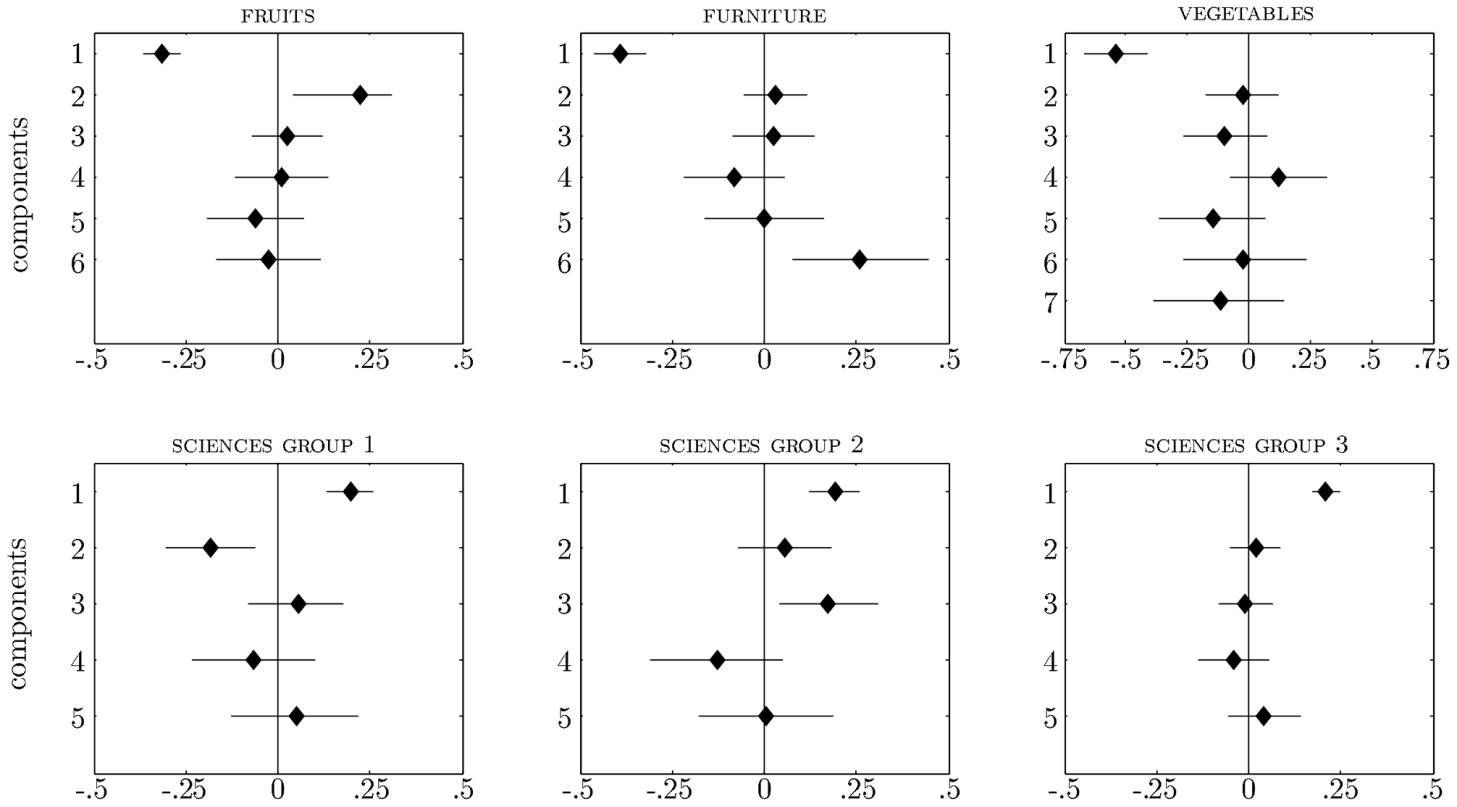
Effects of principal components on item organization as indicated by regression weights for the three single-group categories (upper half) and the one three-groups category (lower half). Horizontal lines indicate the 95% HDI of the posterior of the components' regression weights. Diamonds indicate the regression weights' posterior means.

**Figure 3:**
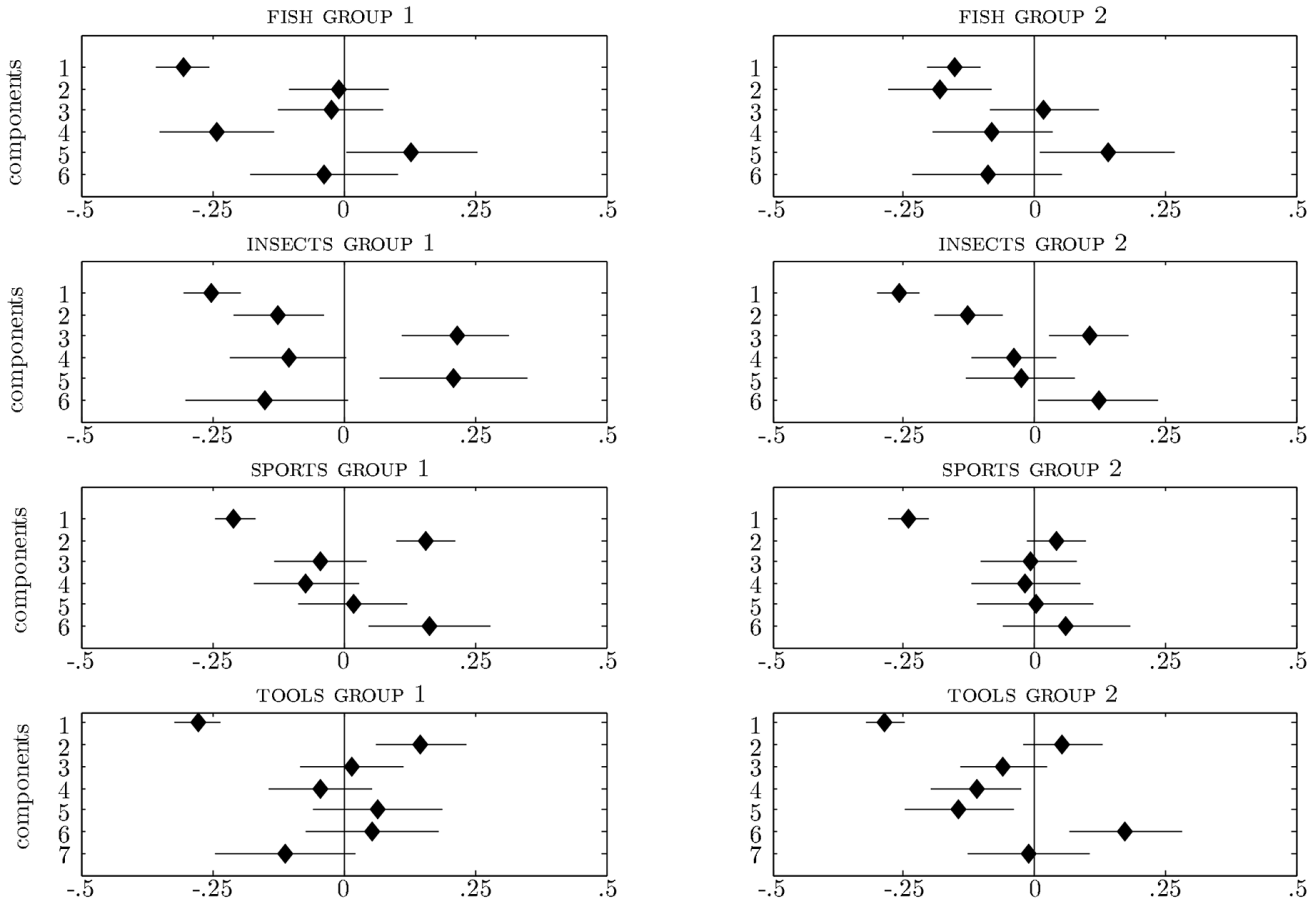
Effects of principal components on item organization as indicated by regression weights for the four two-groups categories. Horizontal lines indicate the 95% HDI of the posterior of the components' regression weights. Diamonds indicate the regression weights' posterior means.

Figures 2 and 3 show the results of these regression analyses. The horizontal lines indicate the 95% HDI of the posterior of the components' regression weights. The diamonds indicate the regression weights' posterior means. The vertical line is the value 0. Components whose 95% HDI does not intersect with the vertical 0 line have a credible nonzero contribution.

For the categories of fruits, furniture, and vegetables there was only one set of 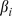 estimates to regress upon the components since the BIC in Study 1 indicated that a one-group solution was to be preferred. In the upper half of Figure 2 there is a subplot representing the regression results for each of these categories. For the category of fruits both Components 1 and 2 had a credible nonzero contribution to 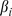. Both Component 1 and Component 6 contributed to 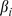 for furniture. For vegetables only Component 1 contributed to 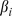. These results are informative as to why some items were considered more likely category members than others. For instance, they indicate that likely fruits <*contain many vitamins*> and <*require a lot of sun*> (features with a strong negative load on the first component). Likely fruits are also <*sweet*>, <*used in desserts*>, and <*grow on trees*> (features loading strongly on the second component). These findings correspond with, for instance, *watermelons* 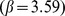 being considered more likely fruits than *onions* 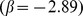.

For the category of sciences three subgroups were identified. The lower half of Figure 2 contains a separate plot representing the regression results for each of the three sets of 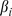 estimates that were retained. The first component had a positive credible nonzero contribution in all three groups. In addition, there was a distinct contribution of Component 2 to the 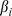 estimates of the first group and a distinct contribution of Component 3 to the 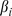 estimates of the second group. A similar pattern emerges for all the categories that had more than one set of 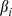 estimates to regress upon the components. For each category, there are one or more components that have a similar effect on every item organization. The first component, which captures the largest proportion of variance in the applicability data, is always among these components. In addition, the item organizations differ with respect to the contribution of one or more other components. The principal components in this manner provide an explanation of the commonalities (in terms of common components) and differences (in terms of distinct components) between the item organizations of different groups. Figure 3 clearly shows this pattern for the remainder of the categories. For each of these categories two subgroups were identified in Study 1.

For the category of fish regression of the first set of 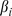 estimates upon the principal components yielded contributions of Components 1, 4, and 5. Regression of the second set of 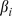 estimates upon the principal components yielded credible contributions of Components 1, 2, and 5. Components 1 and 5 yielded a similar contribution to the two sets of 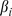 estimates. Different weight was attributed to the information that is conveyed by Components 2 and 4.

Components 1, 2, 3, and 5 predicted the first set of 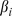 estimates for insects. Components 1, 2, 3, and 6 predicted the mean 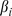 estimates for the second group of categorizers. The categorization decisions of both groups were informed by Components 1, 2, and 3. The information that is conveyed by Components 5 and 6 contributed differently to the categorization decisions in the two groups.

Component 1 was the only one with a credible nonzero contribution to the item organization of the second group for the category of sports, while Components 1, 2, and 6 were found to contribute to the item organization of the first group.

The first component was also the one that yielded a similar contribution to the item organization of the two groups that were obtained for the tools categorization data. It contributed negatively to the items' positions along the dimension of the first group and to the items' positions along the dimension of the second group. There were distinct contributions of Component 2 to the 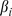 estimates for the first group and of Components 4, 5, and 6 to the 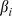 estimates for the second group.

That these results are informative as to why a particular item was a likely member for one group of participants, but was regarded an unlikely category member in another group, can be illustrated for the categories of fish and sports. The components that had a distinct contribution to the two sets of 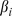 estimates that were retained in each of these categories have a clear interpretation, both in terms of the features and the items that are associated with them. The negative contribution of Component 4 to the 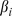 estimates for the first group and of Component 2 to the 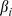 estimates for the second group, for instance, can help explain why *oyster* and *clam* were organized among the non-fish in Group 1 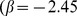 and 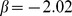, respectively), but were likely fish in Group 2 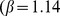 and 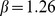, respectively). Both animals scored high on Component 4 (<*is eaten by humans*>) and were the lowest scoring items on Component 2 which appeared to express the shape of prototypical fish with high feature loadings for <*has a tail*> and <*has eyes*>. (Components 1 and 5, which contributed to both sets of 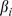 estimates, appeared to convey that fish live under water.)

The regression of the respective 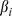 estimates for the sports groups on the principal components reveals why *hiking* 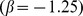 was a less likely category member than *darts* 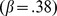 in the first group, but the reverse held in the second group 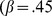 and 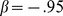, respectively). The first component, which contributed negatively in both groups, could be interpreted as the degree of physical activity involved, with <*burns fat*> as the lowest loading feature. The features <*is played inside*> and <*has rules*> were the ones loading highest on Component 2; <*is performed individually*> was the feature loading highest on Component 6. While *hiking* was deemed the more physical demanding activity, *darts* was the higher scoring item on Components 2 and 6, which contributed positively in the first group.

### Discussion

In Study 2 the distribution of category attributes across candidate items is used to gain insight in the criteria that inform semantic categorization. We don't want to claim that the evaluation of attribute applicabilities is all there is to making categorization decisions (see, for instance, [41] and [42] for complementary accounts of categorization). However, in the current study they do account for a considerable amount of the variability in the items' organization along the latent dimensions (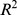 varies between .72 for the second sciences group and .95 for the second tools group). In addition, the results yield a number of sensible observations, both for the single-group categories and the multiple-groups categories.

The results of the regression analyses for the single-group categories (fruits, furniture, vegetables) are insightful in that they demonstrate that there is not just one consideration going into the categorization of items with respect to these target categories. Various components impact the likelihood of category membership and attributes of a varied nature make up these components. One should thus not mistake the retention of one dimension of variation for an argument in favor of a definitional account. Such accounts of category organization have long been discarded [14].

The results for the multiple-groups categories (fish, insects, sports, tools, sciences) corroborate the group differences that were found in Study 1. Subgroups organize items differently with respect to the target category, but also demonstrate substantial agreement on what it means to be a category member. The latter became apparent in Study 1 through the considerable correlations between the 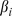 estimates for different groups. In Study 2 it shows in the fact that the same principal component(s) tend to be of importance in all groups. The different subgroups can, however, be distinguished on the basis of other components that are of importance to single groups only. This implies that the subgroups emphasize different sets of category attributes when making their categorization decisions. The idea that distinct groups of categorizers may demonstrate different patterns of attention to and/or weighting of attributes for categorization purposes is not new. The literature on artificial categories contains many illustrations hereof that involve the comparison of groups that are known to differ considerably in their interactions with the category members [43]–[47]. Demonstrations also exist in the literature on natural language categories [22]. Contrary to this, the possibility that an apparently homogeneous sample of participants may be comprised of *latent* subgroups who focus their attention on different aspects of stimuli in order to categorize them, has only been acknowledged in the artificial categories literature [48]–[54]. The current results extend this finding to natural language categories. These results were already anticipated in a threshold theory treatment of inter-individual differences that predicted both thresholds and attribute weights to vary between individuals [55].

Whether the existence of multiple groups of categorizers constitutes evidence for qualitatively different kinds of category representations is a thorny issue (see [43] for a related discussion). The between-group categorization differences that were identified might reflect a fundamentally different representation between groups *or* a shared representation with between-group accentuation differences. In the latter case at least all components in Figures 2 and 3 that showed a credible contribution - regardless of the subgroup for which it was established - are available for consideration when categorizing an item. Individuals from different groups then selectively attend to different components, rendering the weight attributed to the other components effectively zero. Such accentuation differences might result from particular experiences, goals or interests (see [56] for a related illustration). Our current analyses do not empirically distinguish between the two cases. They merely show between-group differences in the kind of information that is used to categorize items. On a related note we need to acknowledge the possibility that the method we have used tends to impose discrete groups on what may in fact be a continuous distribution of component weights within the participant sample. The claim that a difference is discrete rather than continuous is difficult to sustain except as a matter of degree in any case.

## General Discussion

In order to study the nature of the inter-individual differences in semantic categorization, we applied a mixture model to a set of previously published categorization data, pertaining to eight natural language categories of a varied nature (Study 1). The modeling results indicated that the inter-individual categorization differences were not merely differences in the propensity to endorse items as category members (i.e., vagueness), but reflected the use of different criteria for category membership as well (i.e., ambiguity). For the majority of the studied categories, the participant sample fell apart in distinct subgroups that adopted different views on category membership. The various subgroups emphasized different aspects of the multifaceted nature of the categories (Study 2).

These results reveal that our conceptions of familiar natural language categories have a particular kind of ambiguity to them. The identified criteria for category membership do not reflect unrelated senses. Nor do they qualify as facets of words such as *paper* with its [substance] and [print] senses. The alternative interpretations in these kinds of polysemy only share few semantic features [57], [58]. The distinction between the category conceptions of the subgroups is far more subtle. The strong correlation between the different item dimensions (Study 1) and the fact that the sets of principal components predicting them generally only differ by one component (Study 2) constitute evidence for this. It is also not the case that the ambiguity results from contextual differences [59], [60]. The categorization study was conducted in a large auditorium where all participants were present at the same time. Perhaps some of the category terms (e.g., tools) can be best characterized as hyperonyms which subsume a set of microsenses [61]. These have a strong preference for specific use, making a minimal sentence like “Do you possess a tool?” somewhat odd. When confronted with a categorization task without a specific context participants might then adopt either one of a number of available microsenses [62], [63] (but see [11]). Not all of the category terms that we included are subject to this interpretation, however (compare the meaningfulness of “Do you possess a tool?” with that of “Do you possess a fish?”). The ambiguity of these categories is best captured by what are called ways-of-seeing [64]. Ways-of-seeing are modes of construal, similar to different points of view or perspectives. It has been found that depending on the perspective that is taken (e.g., the perspective of faculty members or that of undergraduates) information is differently attended to, yielding category organizations that can differ considerably [65]. Our study differs from this earlier work in a number of important respects. We infer the perspectives from the data, rather than make them explicit beforehand (by asking participants to adopt a certain point of view or by asking members of distinct groups to provide their own point of view). We employ categorization data, while in the earlier work typicality ratings were used. Due to the homogeneous nature of the participant sample and the use of familiar target categories, the different category interpretations we found are also subtler. In [65] participants were apt at taking the category perspective of other groups. It is not clear whether our participants are aware of the different category interpretations or whether they adopt different interpretations on different occasions. Nor is it clear whether they would recognize that individuals are committed to different interpretations. Nevertheless, it is still general practice to rely on raters to establish the number of distinct parts to the meaning of words [66]–[68]. The current study provides one methodological solution for the problem of determining this number for a particular word. The method allows one to infer it from data on the word's extension (see [69] and [70] for alternative methods).

### Improvements over earlier work on natural language categories

The current study of inter-individual differences in semantic categorization constitutes an improvement over earlier approaches that shied away from the possible entanglement of ambiguity and vagueness by assuming that everyone uses the same criteria for category membership. Notable examples include [28] and [29] in which a similar item response theory framework was adopted to study semantic categorization, but only one latent dimension was extracted to account for the categorization performance of all participants. The formal framework we propose here provides for a more flexible tool for understanding the cognitive processes that underlie categorization responses. First, its scope isn't restricted to vagueness (“How many items are endorsed?”), which our results show can yield an overly simple account of semantic categorization differences. Second, categorization strategies (“How are items endorsed?”) may be uncovered using only knowledge of the categorization responses. Choice of strategy needn't be observed. The earlier approaches, on the other hand, require a priori hypotheses or knowledge about subgroups the participant sample might divide in to do so. A separate model analysis of the kind proposed in [28] and [29] could then be undertaken for each of these groups. Evidence for different categorization strategies would only be established if the responses by the known groups yielded distinct item organizations. In addition, such an analysis might overlook important differences between alternative partitions of the participants.

The mixture model's ability to provide a rich account of categorization from mere binary decisions is an asset. It is also rather straightforward to extend the model to use more information. One could include other sources of information about the representativeness of items such as mouse movement trajectories [71] or reaction times [72]. For an example of how to incorporate the latter in a mixture item response theory model see [73]. Taken together, these arguments suggest the proposed model makes for a flexible tool to analyze semantic categorization data. That is not to say that further development of the model is not in order for future successful application. For instance, in the current form of the model it is assumed that an individual applies the same considerations for categorization towards all candidate items. However reasonable this assumption might be for an individual who is presented with a short list of items to work through, it is always possible to conceive of items and/or situations that might violate this assumption.

### Improvements over earlier work on artificial categories

The current study also illustrates an alternative for present treatments of inter-individual differences in artificial category learning studies. It is now widely acknowledged that all too often in the artificial category learning literature individual differences have been disregarded in favor of a treatment of the ‘average’ categorizer [74], [75]. Averaging across categorizers might lead one to infer cognitive processes that do not characterize any of the individual categorizers. Moreover, it is doubtful that a sole focus on how individuals are the same will provide a representative account of the complexities of human behavior, like categorization. A focus on how individuals are different carries the opposite risk of an overly complicated account of the phenomenon under study [32], [76]. Providing a separate account of the data of each additional categorizer is not necessarily very informative. It might identify processes that have already been found in other individuals and is subject to noise-fitting. It appears that an intermediate account that expresses how individuals are both the same and different is to be preferred.

Our treatment of inter-individual differences in categorization offers such an intermediate account. It expresses how individuals are the same by placing them in a subgroup of categorizers who all employ the same criteria for category membership. (One set of 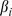 estimates is extracted for each subgroup.) It expresses how they are different in two manners. First, individuals can be placed in discrete subgroups that display distinct categorization behavior. Second, within each of the identified subgroups inter-individual differences in the placement of the categorization cut-off are allowed. (A 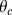 estimate is extracted for every categorizer.) These continuous differences set our framework apart from formal accounts of artificial category learning that take individuals within a latent group to be invariants [48], [52], [77], [78]. When subgroups of similarly behaving participants are identified and the data from the individuals within a group is subsequently aggregated, different parameterizations of the employed formal account can signal the manners in which the subgroups differ from one another. However, such an approach only allows for discrete differences. There is now convincing evidence that there are data in the artificial category learning literature that warrant a formal treatment that involves both discrete and continuous inter-individual differences, similar to ours [49].

The usefulness of the idea that individuals may fall in a number of distinct classes with some degree of individual variation in each need not be restricted to the study of categorization in natural language categories and artificial categories. There is a now growing acknowledgment that several cognitive behaviors present with both discrete (between-group) and continuous (within-group) inter-individual differences (see [79] and [80] for notable examples in the fields of decision making and conditional reasoning, respectively). It is our conviction that many more exist that can benefit from the approach that we have outlined in this paper.
